# Illustration of the gut-immune-liver axis in early-stage alcohol-related liver disease: role and non-invasive potentiation of biomarkers and peptidomic signatures

**DOI:** 10.1038/s44355-026-00072-0

**Published:** 2026-06-30

**Authors:** Anjali Kumari, Mukesh Sriwastva, Michael Merchant, Christine Dolin, Gavin Arteel, Sabine Waigel, Maiying Kong, Melanie L. Schwandt, Joris Verster, Ashwani K. Singal, Venkatakrishna R. Jala, Craig J. McClain, Vatsalya Vatsalya

**Affiliations:** 1 Division of Gastroenterology, Hepatology and Nutrition, Department of Medicine, University of Louisville, Louisville, KY USA; 2 Clinical Laboratory for the Intervention Development of AUD and Organ Severity, University of Louisville, Louisville, KY USA; 3 Department of Physiology, University of Louisville, Louisville, KY USA; 4 Div. of Nephrology and Hypertension, Department of Medicine, Louisville, KY USA; 5 Genomics Training and Education Center, University of Louisville, Louisville, KY USA; 6 Department of Bioinformatics and Biostatistics, University of Louisville, Louisville, KY USA; 7 National Institute on Alcohol Abuse and Alcoholism, Bethesda, MD USA; 8 Utrecht University, Utrecht, Holland; 9 Robley Rex VA Medical Center, Louisville, KY USA; 10 University of Louisville Alcohol Research Center, Louisville, KY USA; 11 Department of Microbiology and Immunology, UofL, Louisville, KY USA

**Keywords:** Biochemistry, Biomarkers, Diseases, Gastroenterology, Immunology

## Abstract

Alcohol use disorder (AUD) can progress to alcohol-associated liver disease (ALD), yet early-stage ALD (eALD) remains poorly defined at the molecular level. We performed plasma proteomic and peptidomic analysis in 42 adults (AUD *n* = 10, eALD *n* = 26, healthy volunteers *n* = 6) using LC-MS/MS and pathway enrichment. Individuals with eALD exhibited greater alcohol burden, elevated liver injury markers (AST and ALT), and increased gut–immune dysfunction markers, including lipopolysaccharide, TNF-α, and K18M65, compared with AUD. Proteomic profiling identified 23 dysregulated peptides, with fibrinogen alpha (FIBA), fibrinogen beta (FIBB), COL1A1, and ITIH4 as key candidates. FIBB and COL1A1 were associated with AST, while FIBA and COL1A1 distinguished eALD from AUD. These findings identify circulating peptide biomarkers reflecting gut–immune–liver axis dysfunction and support their potential utility for non-invasive detection of early alcohol-associated liver disease.

## Introduction

Alcohol use disorder (AUD) and alcohol-associated liver disease (ALD) are major contributors to morbidity and mortality globally^1,2^. ALD is now recognized as a leading cause of liver-related deaths worldwide^3^. Disease progression is often insidious, with many individuals remaining asymptomatic until advanced stages, when damage may become irreversible^4^. The concept of early-stage ALD (eALD) has been increasingly emphasized. In clinical studies, eALD is typically defined by elevated liver enzymes indicating hepatocellular injury in individuals with chronic alcohol exposure, but without evidence of cirrhosis or advanced fibrosis. It refers to the early stage of alcohol-associated liver injury that occurs before significant cirrhosis develops^5^. Early identification of ALD is crucial, as this window offers the best chance for intervention through alcohol abstinence and targeted therapy, increasing the potential to halt or reverse disease progression and lessening the undesirable impact of more severe liver disease^6^.

Alcohol increases gut permeability, allowing bacterial byproducts (endotoxins and metabolites)^7^ to reach the liver via the portal vein, triggering the activation of liver macrophages and Kupffer cells, and the release of proinflammatory cytokines, including TNF-α, IL-6, and IL-8^8^. Simultaneously, alcohol metabolites cause hepatocyte oxidative stress, mitochondrial dysfunction, and cell death, further promoting steatosis^9,10^. Historically, the diagnosis and monitoring of ALD have relied on traditional liver enzyme tests such as ALT, AST, and GGT^11–13^. However, these tests lack sensitivity and specificity in detecting early-stage disease^14–16^. Although biopsy remains the gold standard for diagnosis, its invasive nature and associated risks limit its utility, especially before advanced liver damage is evident^17^.

Technological advances, especially in mass spectrometry-based proteomics, offer new opportunities to characterize disease-associated molecular signatures in plasma^18,19^. Identifying novel biomarkers could significantly enhance diagnostic accuracy, enable earlier intervention, and improve outcomes in individuals with ALD^20,21^. Despite advances in understanding ALD, early-stage disease remains poorly characterized at the molecular level, particularly with respect to integrated gut–immune–liver axis dysfunction^22^. Most prior studies have focused on advanced disease or single biomarker classes, limiting the ability to capture early, multi-compartment biological changes. In particular, the role of circulating peptidomic signatures as early indicators of coordinated gut barrier disruption, immune activation, and hepatic injury remains underexplored.

In the present study, we perform an integrated clinical, inflammatory, and plasma peptidomic analysis in individuals with AUD with and without early-stage liver injury. By combining proteomic profiling with clinical and biomarker correlations, we identify candidate peptide signatures linked to key pathophysiological processes across the gut–immune–liver axis. This approach enables the characterization of early molecular alterations preceding advanced disease and provides a framework for non-invasive biomarker development in ALD.

## Results

### Cohort characteristics and clinical features

To characterize clinical differences between groups, we analyzed demographic, drinking history, and biochemical parameters. A total of 42 participants were included, comprising healthy volunteers (HV, *n* = 6), individuals with AUD without liver injury (AUD, *n* = 10), and individuals with AUD with early-stage alcohol-associated liver disease (eALD, *n* = 26).

The mean ages of the AUD and eALD groups were comparable (approximately 42.6 years), whereas HV participants were younger. Body mass index (BMI) values in both AUD and eALD groups were within the overweight range, while HV participants had BMIs within the normal range. Due to the limited number of males in AUD, sex-based comparisons were interpreted cautiously.

Drinking behavior analysis indicated a higher overall alcohol burden in the eALD group. Participants in eALD reported greater total alcohol consumption in the past 90 days (TD90) and a higher number of heavy drinking days (HDD90) compared with AUD. The number of non-drinking days (NNDD90) was lower in eALD, whereas the average drinks per drinking day (AvgDPD90) were similar between groups (~14 drinks per drinking day). Lifetime drinking history (LTDH) was significantly higher in the eALD group compared with AUD (*p* = 0.028).

Biochemical markers of liver injury were significantly elevated in eALD. Aspartate aminotransferase (AST) levels were approximately threefold higher in eALD compared with AUD (*p* = 0.013), and alanine aminotransferase (ALT) levels were approximately fourfold higher in eALD compared with both AUD and HV participants (*p* < 0.001). Plasma lipopolysaccharide (LPS) levels were also significantly increased in eALD. Albumin concentrations were modestly lower and statistically significant in eALD compared with AUD (*p* = 0.037). The AST:ALT ratio did not differ significantly between groups.

Sex-related differences were observed in liver enzyme profiles. eALD males exhibited higher AST levels than eALD females, and eALD females had higher AST levels than AUD females. ALT levels were significantly elevated in eALD compared with AUD in both sexes. Overall, most participants with AUD and eALD had elevated BMI, suggesting a contribution of metabolic factors to hepatic injury. Detailed cohort characteristics are summarized in Table 1.

**Table 1:** Demographic, drinking history, and liver injury characteristics of study participants

Measures	Group 1			Group 2			Healthy volunteers	Across groups *p* value
	Males (*n* = 4; 40%)	Females (*n* = 6; 60%)	Total (*n* = 10; 23.81%)	Males (*n* = 20; 76.92%)	Females (*n* = 6; 23.08%)	Total (*n* = 26; 61.9%)	Total (*n* = 6)	
Baseline demographics								
Age (years)	42.9 ± 11.9	42.4 ± 14.6	42.6 ± 12.68	42 ± 9.12	44.6 ± 11.45	42.6 ± 9.52	27.83 ± 4.66	0.007
BMI	26.7 ± 2.73	29.2 ± 11.88	28.2 ± 9.09	26.41 ± 4.27	26.87 ± 2.74	26.52 ± 3.91	23.56 ± 2.54	NS
Drinking history								
TD90	972.65 ± 330.29	921.3 ± 295.86	944.1 ± 527.62	1159.76 ± 482.56	855.83 ± 410.47	1123.32 ± 470.38	43 ± 37.8	<0.001
HDD90^d=0.049^	73 ± 16.06	52.00 ± 18.41	61.33 ± 19.72	71.00 ± 22.84	77.17 ± 17.82	72.42 ± 21.61	3.33 ± 5.05	<0.001
AvgDPD90^b=0.037^	12.73 ± 3.57	16.24 ± 8.3	14.68 ± 6.90	15.46 ± 4.74	10.51 ± 4.28	14.32 ± 5.08	1.55 ± 0.46	<0.001
NNDD90^d=0.040^	12.25 ± 18.73	35.80 ± 18.12	25.33 ± 21.25	14.80 ± 19.13	10.67 ± 14.99	13.85 ± 18.06	63.00 ± 23.39	<0.001
LTDH^c*=0.055*^	10 ± 6.53	9.17 ± 5.78	9.5 ± 5.74	19.37 ± 8.98	11.5 ± 8.98	17.48 ± 10.46	1.55 ± 0.4	0.040
Liver injury markers								
AST (IU/L)^b = 0.002, c = 0.053, d = 0.016^	38.13 ± 6.53	32.33 ± 16.31	36.9 ± 21.96	89.4 ± 63.59	220.83 ± 130.05	119.73 ± 98.21	21.25 ± 4.81	0.011
ALT (IU/L)^a = 0.023, c < ^^0.001, d = 0.047^	32.5 ± 7.33	21.17 ± 5.53	25.7 ± 8.31	85.65 ± 107.35	136.17 ± 107.35	97.31 ± 59.8	25.00 ± 2.54	<0.001
AST/ALT^b = 0.006^	1.32 ± 0.79	1.48 ± 0.42	1.42 ± 0.56	1.00 ± 0.51	1.91 ± 1.01	1.21 ± 0.75	1.23 ± 0.29	NS
Total bilirubin (mg/dL)	0.75 ± 0.27	1.07 ± 1.46	0.94 ± 1.11	0.57 ± 0.37	0.75 ± 0.42	0.61 ± 0.38	0.55 ± 0.11	NS
Albumin (g/dL)	3.85 ± 0.19	3.95 ± 0.41	3.91 ± 0.32	4.14 ± 0.39	4.22 ± 0.36	4.14 ± 0.37	4.03 ± 0.35	NS

*BMI* body mass index, *TD90* total drinks in the past 90 days, *HDD90* heavy drinking days in the past 90 days, *AvgDPD90* average drinks per drinking day, *NNDD90* number of non-drinking days, *LTDH* lifetime drinking history, *AST* aspartate aminotransferase, *ALT* alanine aminotransferase. Values are presented as mean ± standard deviation. *NS* indicates not significant. Superscripts denote subgroup comparisons as described in the table.

### Gut barrier dysfunction and inflammatory activation

To assess gut–immune alterations, we examined markers of microbial translocation, inflammation, and hepatocyte injury. Plasma LPS concentrations were approximately twofold higher in eALD compared with AUD (*p* = 0.001), indicating increased gut permeability. Additional gut-derived markers, including lipopolysaccharide-binding protein (LBP), soluble CD14 (sCD14), and calprotectin, were numerically increased in eALD but did not reach statistical significance.

LBP levels were positively associated with total alcohol consumption over 90 days (TD90) in the eALD group (*p* = 0.045). sCD14 levels were significantly higher in females compared with males within the eALD group (*p* = 0.033).

Consistent with increased endotoxin exposure, inflammatory cytokine analysis demonstrated elevated tumor necrosis factor-α (TNF-α) levels in eALD compared with AUD (*p* = 0.022). Other cytokines, including IL-6, IL-8, IL-1β, and MCP-1, showed numerical increases. Within the eALD group, AST levels were significantly associated with IL-1β and TNF-α (*p* = 0.039), linking inflammatory activation to hepatocellular injury.

Markers of hepatocyte injury were also increased in eALD. Cytokeratin-18 M65 (K18M65) levels were significantly higher in eALD compared with AUD (*p* = 0.04). The K18 (M65/M30) ratio was elevated in eALD and was significantly associated with the AST:ALT ratio (adjusted *R*² = 0.218, *p* = 0.009). These results demonstrate concurrent elevations in gut barrier dysfunction, inflammatory activation, and hepatocyte injury in eALD (Table 2).

**Table 2:** Markers of gut dysfunction, inflammation, and hepatocyte injury in AUD and early alcohol-associated liver disease

Measures	Group 1 (*n* = 10; 23.81%)	Group 2 (*n* = 26; 61.9%)	Between groups *P* value
Gut dysfunction biomarkers			
Calprotectin (µg/g)	234.91 ± 110.81	308.94 ± 155.86	NS
Flagellin (ng/mL)	4.36 ± 5.23	4.27 ± 3.21	NS
LPS (ng/mL)	0.05 ± 0.02	0.10 ± 0.07	0.001
LBP (ng/mL)	1669.70 ± 2859.71	2092.84 ± 3176.35	NS
sCD14 (ng/mL)	9397.99 ± 1656.43	9642.75 ± 1761.82	NS
TF-3 (ng/mL)	1.06 ± 0.91	0.92 ± 1.25	NS
Immune response and inflammation biomarkers			
IgM (mg/dL)	132.7 ± 67.87	110.27 ± 45.08	NS
PAI-1 (ng/mL)	40.41 ± 12.43	46.35 ± 28.59	NS
IgG (mg/dL)	1277.3 ± 401.9	1060.9 ± 324.98	NS
Interleukin-6 (pg/mL)	2.96 ± 3.69	4.04 ± 3.35	NS
Interleukin-8 (pg/mL)	11.15 ± 22.08	6.87 ± 11.14	NS
IL-1β (pg/mL)	0.42 ± 0.32	0.48 ± 0.26	NS
Adiponectin (µg/mL)	32.47 ± 25.11	28.78 ± 17.20	NS
TNF-α (pg/mL)	1.44 ± 0.68	2.15 ± 0.95	0.022
Leptin (pg/mL)	7683.88 ± 8555.32	5444.37 ± 5362.86	NS
MCP-1 (pg/mL)	103.73 ± 56.39	120.03 ± 68.12	NS
Liver cell death biomarkers			
M65 (U/L)	381.6 ± 484	924.3 ± 1028	0.04
M30 (U/L)	457.0 ± 666	399.3 ± 395.4	NS
K18Ratio	1.21 ± 0.82	2.15 ± 1.26	0.016
Nutritional status			
CONUT (unit score)	1.3 ± 1.56	0.92 ± 1.05	NS

*CONUT* controlling nutritional status, *IgG* immunoglobulin G, *IgM* immunoglobulin M, *LBP* lipopolysaccharide-binding protein, *LPS* lipopolysaccharide, *M65* total cytokeratin-18, *M30* caspase-cleaved cytokeratin-18, *MCP-1* monocyte chemoattractant protein-1, *PAI-1* plasminogen activator inhibitor-1, *sCD14* soluble CD14, *TF-3* trefoil factor 3, *TNF-α* tumor necrosis factor-α. Values are presented as mean ± standard deviation. *P* values correspond to comparisons between groups.

### Plasma peptidomic profiling

To identify molecular signatures of early disease, plasma peptidomic profiling was performed. A total of 5370 peptides were detected across all samples. Differential expression analysis revealed significant alterations between study groups, with 95 peptide accessions differing between AUD and eALD.

Quantitative analysis identified 23 significantly dysregulated proteins in eALD, including 19 upregulated and four downregulated candidates. Key proteins included fibrinogen alpha (FIBA), fibrinogen beta (FIBB), collagen type I alpha 1 (COL1A1), hematopoietic cell-specific Lyn substrate 1 (HCLS1), and inter-alpha-trypsin inhibitor heavy chain 4 (ITIH4).

**Fig. 1:**
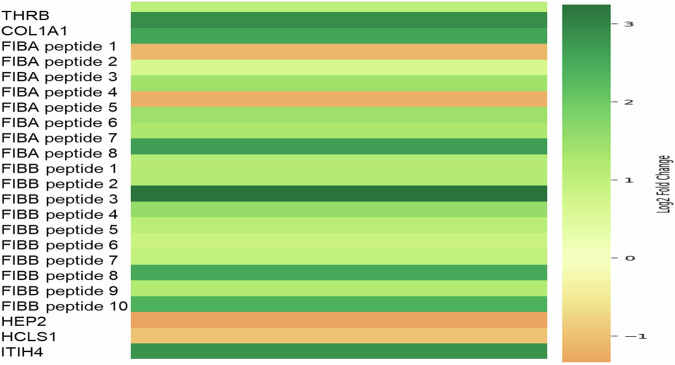
Differential plasma peptide expression in early alcohol-associated liver disease. Heatmap showing log2 fold changes of selected peptides, including THRB, COL1A1, FIBA, FIBB, HEP2, HCLS1, and ITIH4. Green indicates increased expression and orange indicates decreased expression, with color intensity reflecting magnitude. All displayed peptides met statistical significance (*p* < 0.05). Multiple entries for fibrinogen peptides represent distinct proteoforms identified by unique peptide sequences.

Heatmap visualization demonstrated distinct peptide abundance patterns across groups, with increased expression of fibrinogen-related peptides and extracellular matrix components in eALD (Fig. 1). Differential expression was defined as *p* < 0.05 after false discovery rate correction.

### Association of peptides with clinical biomarkers

**Fig. 2:**
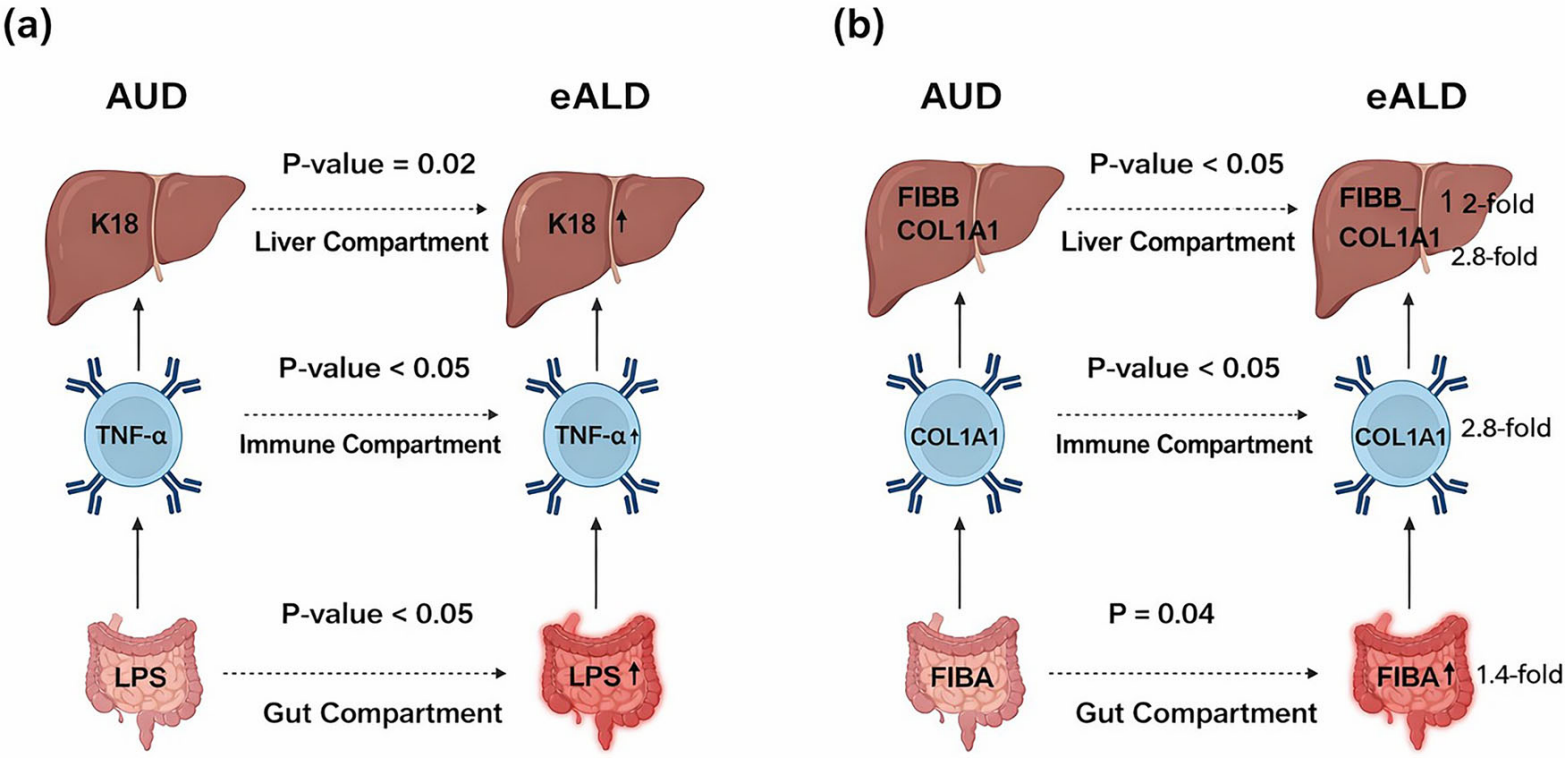
Gut–immune–liver axis alterations and peptidomic integration in early alcohol-associated liver disease. **a** A schematic representation of clinical biomarker changes across the gut, immune, and liver compartments comparing alcohol use disorder (AUD) and early alcohol-associated liver disease (eALD). **b** Integrated peptidomic alterations across compartments in AUD and eALD. Directional arrows indicate increased biomarker and peptide abundance across compartments, highlighting coordinated dysregulation of gut permeability, inflammatory signaling, and hepatic injury in early disease.

To evaluate biological relevance, we examined associations between peptide abundance and clinical biomarkers. Peptide expression patterns differed between groups, with increased abundance of fibrinogen-derived peptides and extracellular matrix proteins in eALD (Fig. 2).

**Fig. 3:**
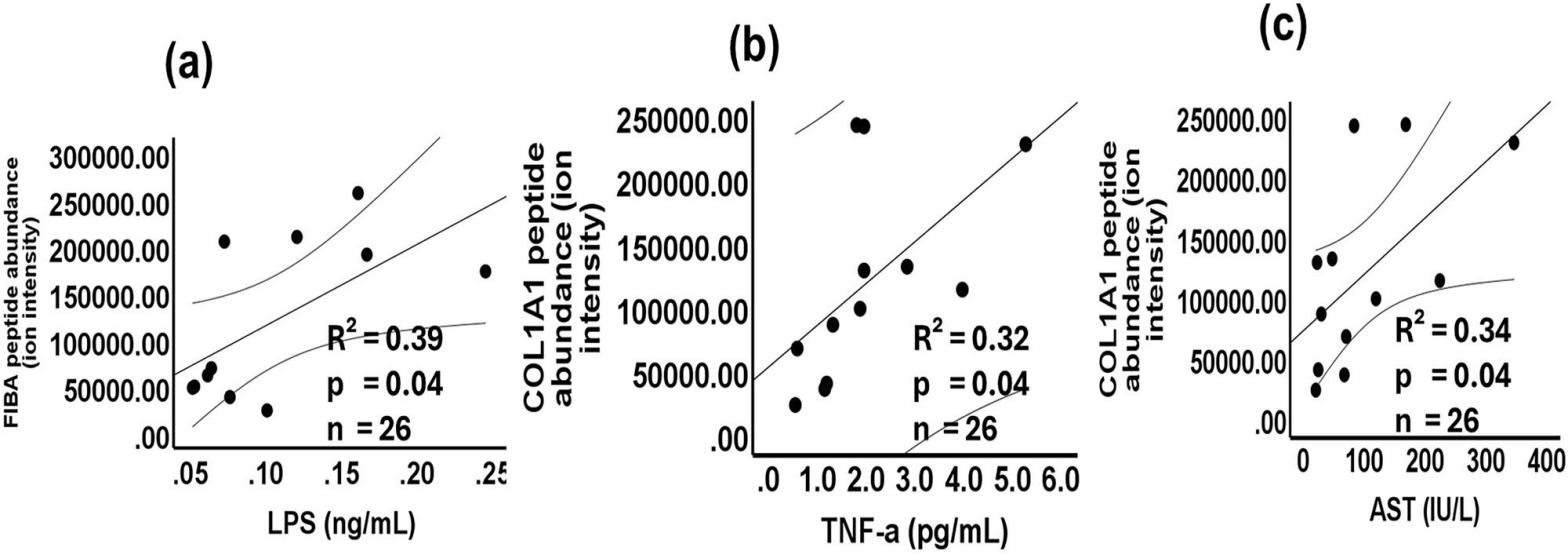
Associations between peptide abundance and clinical biomarkers. **a** Relationship between FIBA peptide abundance and plasma LPS levels. **b** Association between COL1A1 peptide abundance and TNF-α concentrations. **c** Association between COL1A1 peptide abundance and AST levels. Solid lines represent linear regression fits, and shaded areas indicate 95% confidence intervals.

Significant compartment-specific associations were observed. FIBA abundance was positively associated with plasma LPS levels (*R*² = 0.39, *p* = 0.04), linking peptide expression to gut permeability. COL1A1 abundance was associated with TNF-α levels (*R*² = 0.32, *p* = 0.04) and AST levels (*R*² = 0.34, *p* = 0.04), indicating relationships with inflammation and liver injury (Fig. 3a–c).

**Fig. 4:**
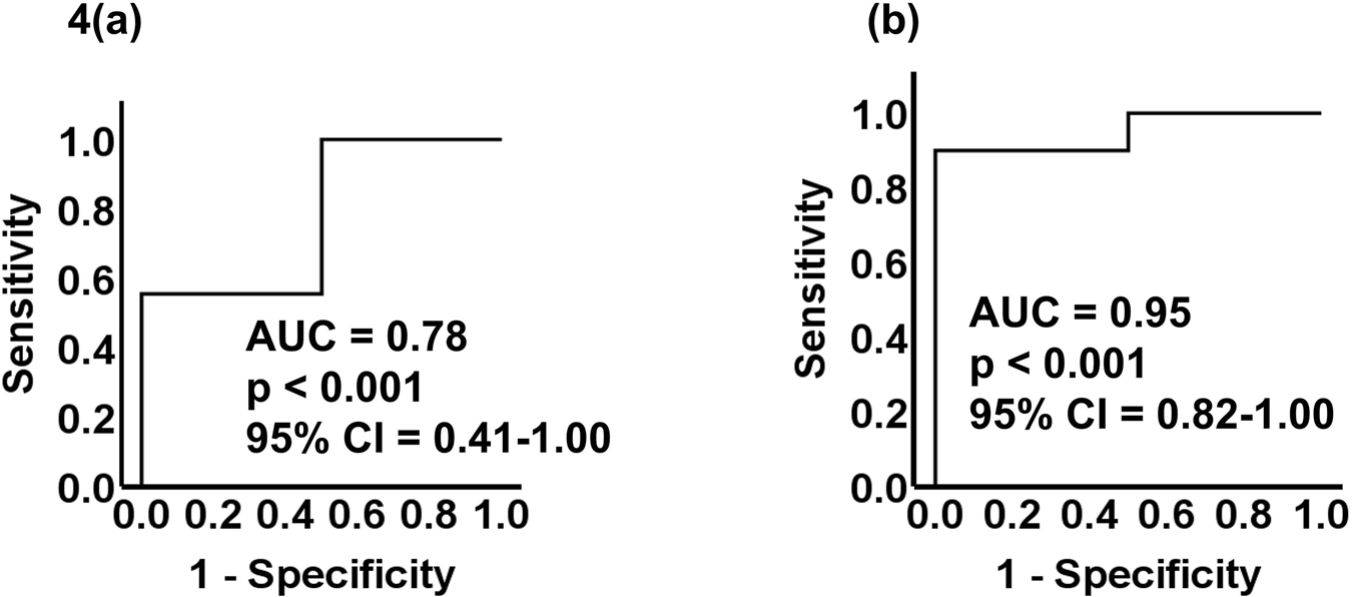
Diagnostic performance of candidate peptide biomarkers. Receiver operating characteristic (ROC) curves evaluating discrimination between early alcohol-associated liver disease and alcohol use disorder. **a** FIBA peptide showing moderate diagnostic performance. **b** COL1A1 peptide demonstrating strong discriminatory capability. Sensitivity is plotted against one minus specificity.

Receiver operating characteristic analysis demonstrated the ability of candidate peptides to distinguish eALD from AUD. Given the exploratory pilot design and limited sample size, these estimates should be interpreted as preliminary and require validation in independent cohorts. The FIBA peptide showed moderate diagnostic performance (AUC = 0.78), whereas COL1A1 demonstrated strong discrimination (AUC = 0.95), although these findings require validation in larger cohorts (Fig. 4a, b).

**Fig. 5:**
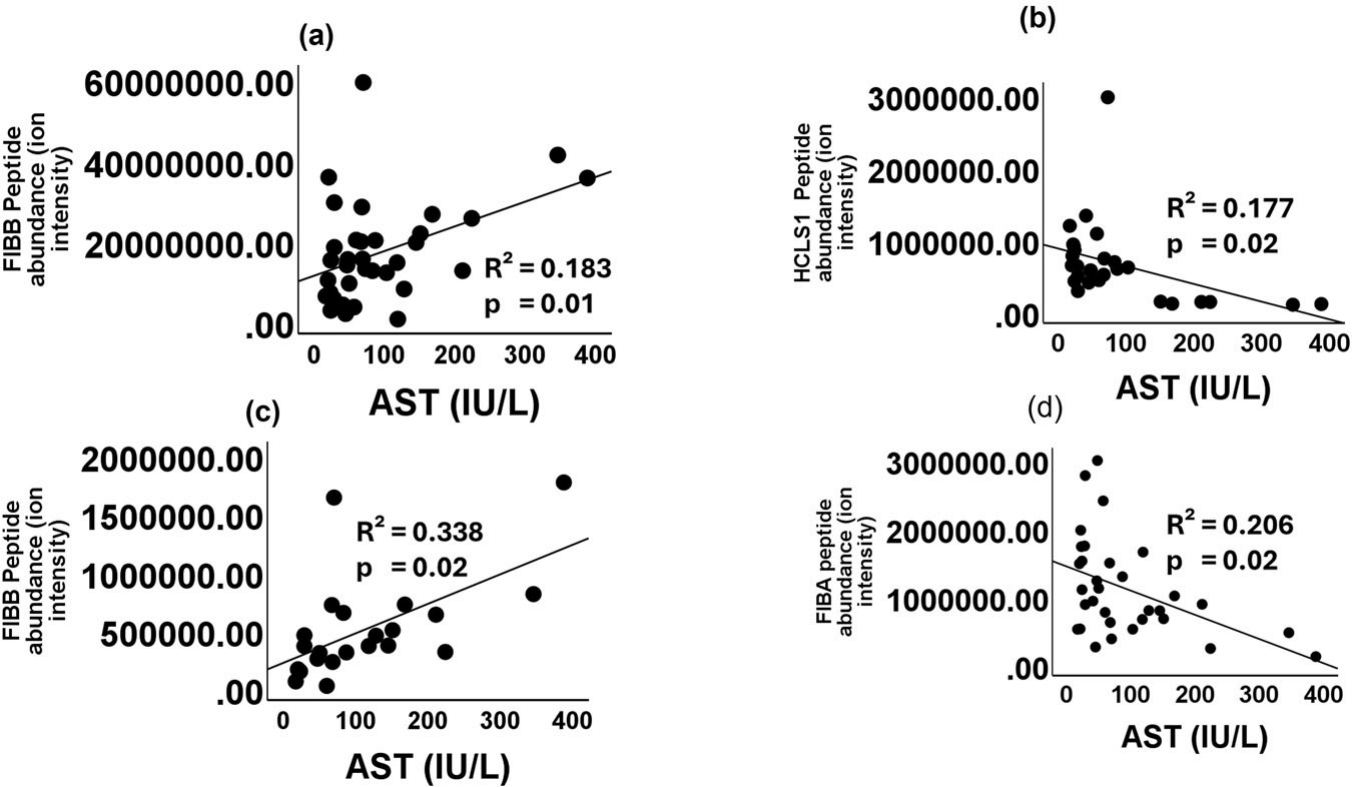
Correlation between peptide biomarkers and liver injury. **a**–**d** Scatter plots showing relationships between peptide abundance and AST levels. Linear regression lines illustrate the direction and strength of associations.

Associations with AST were further evaluated. FIBB peptide abundance was positively correlated with AST levels (*R*² = 0.183, *p* = 0.01), whereas HCLS1 showed a negative association (*R*² = 0.177, *p* = 0.02). A second FIBB peptide demonstrated a stronger positive correlation (*R*² = 0.338, *p* = 0.02), while FIBA showed an inverse association with AST (*R*² = 0.206, *p* = 0.02). These results demonstrate that peptide signatures track with biochemical measures of liver injury (Fig. 5a–c).

### Network analysis of protein interactions

To explore pathway-level organization, protein–protein interaction analysis was performed. The resulting network revealed a highly interconnected structure centered on fibrinogen components and thrombin signaling. The network included seven nodes and 11 edges, with an average node degree of 3.14 and a clustering coefficient of 0.8. Interaction enrichment was statistically significant (PPI*p* = 8.28 × 10⁻¹⁰).

**Fig. 6:**
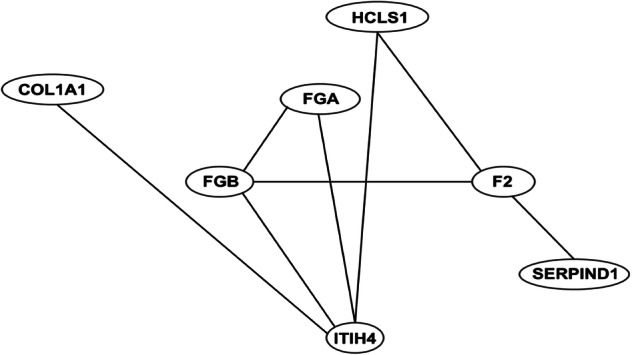
Protein–protein interaction network in early alcohol-associated liver disease. Network analysis illustrating interactions among key proteins involved in coagulation and extracellular matrix remodeling. Central nodes include fibrinogen components and thrombin, indicating coordinated activation of prothrombotic and fibroinflammatory pathways.

Central nodes included thrombin (F2), fibrinogen alpha (FGA), and fibrinogen beta (FGB), indicating activation of prothrombotic pathways. Peripheral nodes, including COL1A1 and ITIH4, linked extracellular matrix remodeling with inflammatory signaling. These results demonstrate coordinated activation of coagulation and fibroinflammatory pathways in eALD (Fig. 6).

## Discussion

In this study, we provide an integrated characterization of eALD by combining clinical phenotyping, inflammatory profiling, and plasma peptidomic analysis. Our findings demonstrate that early disease is associated with coordinated alterations across the gut–immune–liver axis, reflected by increased microbial translocation, systemic inflammatory activation, and distinct circulating peptide signatures. Importantly, these changes are detectable prior to advanced fibrosis or cirrhosis, highlighting the presence of measurable molecular perturbations in early disease states.

Elevated plasma LPS levels observed in eALD support the presence of increased gut permeability, a well-established feature of ALD^23,24^. This endotoxemia is known to activate innate immune pathways, including Toll-like receptor signaling, leading to increased production of proinflammatory cytokines such as TNF-α, which contribute to hepatocellular injury^25,26^. The observed associations between inflammatory mediators and liver injury markers in our cohort further support this mechanistic framework.

A key strength of this study is the integration of multi-compartment biology through circulating peptide signatures. While prior studies have independently examined gut permeability, inflammatory mediators, or proteomic alterations, few have linked these processes within a unified framework in early-stage disease. Our results suggest that fibrinogen-derived peptides and extracellular matrix components, such as COL1A1, may serve as integrative biomarkers reflecting both systemic inflammation and early tissue remodeling. This supports the concept that circulating peptides can capture complex pathophysiological interactions rather than isolated biological processes.

Proteomic analysis identified significant alterations in fibrinogen-related peptides (FIBA and FIBB) and extracellular matrix-associated proteins such as COL1A1. These findings suggest early activation of coagulation and fibrogenic pathways, which are increasingly recognized as key components of liver disease progression^27,28^. The increased abundance of fibrinogen-derived peptides may reflect systemic inflammatory and prothrombotic responses, while elevated COL1A1 levels may indicate early extracellular matrix remodeling.

Importantly, several candidate peptides demonstrated associations with clinical indicators of liver injury. COL1A1 abundance was associated with both TNF-α levels and AST concentrations, linking extracellular matrix remodeling with inflammatory activation and hepatocellular injury. Similarly, fibrinogen-derived peptides were associated with AST, supporting their potential role as markers of early liver injury. Although the observed correlations were modest, this is expected in early-stage human disease, where biological variability is high and pathological changes are still evolving. Similar modest but biologically meaningful associations have been reported in early alcohol-associated and metabolic liver disease^29,30^.

Network analysis further highlighted the integration of coagulation and extracellular matrix pathways in eALD. The identification of a highly interconnected network centered on fibrinogen components and thrombin signaling suggests coordinated activation of prothrombotic and fibroinflammatory pathways. These findings align with emerging evidence linking coagulation, inflammation, and tissue remodeling in liver disease progression^31–33^.

From a translational perspective, circulating peptide biomarkers identified in this study may provide a non-invasive approach for early detection of ALD. Current diagnostic strategies rely heavily on liver enzymes, which lack sensitivity and specificity, or liver biopsy, which is invasive and not suitable for early-stage screening. The observed discriminatory performance of candidate peptides such as COL1A1 supports their potential integration into clinical risk stratification frameworks. Although these findings are preliminary and require validation in larger cohorts, they highlight the feasibility of leveraging circulating peptidomic signatures to detect early molecular alterations preceding advanced liver injury.

## Methods

### Patient recruitment

This study was conducted in accordance with the Declaration of Helsinki and was approved by the Central NeuroScience Committee of the IRB, NIAAA, NIH, Bethesda, MD (Protocol: 98-AA-0009) for the AUD and eALD patients' cohort; and Institutional Review Board of the University of Louisville, KY, USA (IRB approval number 12.0427) for HV cohort. All participants provided written informed consent prior to enrollment. This clinical study was registered at ClinicalTrials.gov (NCT00106106) on 3-19-2005. Patients enrolled had a diagnosis of AUD according to the DSM-IV, based on the alcohol dependence module of the SCID I-interview. Additional information on patient participation and enrollment can be obtained from previous studies^3,4,6^. All AUD and eALD patients were consented and enrolled in this study at the NIAAA Clinical Center, and HV at the University of Louisville, before collecting clinical and research data and bodily samples.

eALD was defined clinically by elevated aminotransferases in individuals with AUD, in the absence of advanced fibrosis, cirrhosis, or hepatic decompensation. This definition aligns with current clinical and histopathological frameworks describing early alcohol-related liver injury.

### Participants were classified as eALD if they had

(i) Documented chronic alcohol consumption consistent with AUD;
(ii) Biochemical evidence of liver injury defined by elevated aminotransferases (AST and/or ALT above the upper limit of normal, ULN); and
(iii) Absence of advanced fibrosis, cirrhosis, or hepatic decompensation based on clinical evaluation and available diagnostic assessment.

The present study was designed as an exploratory pilot study aimed at identifying candidate plasma proteomic and peptidomic signatures associated with eALD. Given the challenges in recruiting early-stage clinical cohorts, the study was intended for hypothesis generation and biomarker discovery rather than definitive validation.

### Eligibility criteria

The inclusion criteria required for eALD participants to have elevated liver enzymes (alanine transaminase, ALT > 40 IU/L) and AST:ALT was <1.5, as per the eALD presentation criteria.

All participants were screened for underlying liver diseases using clinical history, serological testing, and predefined inclusion criteria; however, imaging was not performed in all participants due to protocol-related resource limitations. None of the enrolled participants was actively receiving pharmacologic treatment for AUD (e.g., naltrexone, disulfiram) at the time of sample collection. Baseline heavy drinking biomarkers were elevated in all patients. They also had a positive, clinically significant baseline craving score and positive heavy alcohol drinking patterns for the past 90 days (Table 1)^11^, in addition to meeting the clinical diagnostic criteria for AUD^34^. The patients had an average of 18 years of drinking duration. The patients drank 14 drinks per day on average and reported 66 heavy drinking days per 90 days on average as well. The participants were between 18 and 58 years of age. Individuals were excluded if they had a history of severe alcohol withdrawal, active psychiatric or neurological disorders (excluding AUDs), current use of contraindicated medications, severe renal or hepatic dysfunction, pregnancy, or breastfeeding.

### Clinical and research data

Thirty-six enrolled study participants were grouped categorically using clinical levels of alanine transaminase (ALT > 40 IU/L) from the routine liver injury panel of Biochemistry 20 testing. Clinical data and blood samples were collected during enrollment. Demographics (age, sex, BMI) and recent drinking history information were also recorded at the time of the study visit. The 90-day timeline follows back^31^ (TLFB90: TD90, NDD90, AvgDPD90, HDD90) for the recent drinking profile, LTDH^35^.

Recent alcohol drinking and drinking patterns of drinking were collected from the timeline follow-back questionnaire^36^, a self-reported inventory of alcohol intake collected in the clinical setting under clinical supervision. Measures assessed for the past 90 days included Total Drinks (TD90), Number of Drinking Days (NDD90), Number of Non-Drinking Days (NNDD90), Average Drinking per day (AvgDPD90), and Heavy Drinking Days (HDD90). Chronic drinking (number of years) was assessed using the LTDH (in years) questionnaire^37^. We also used the “Controlling Nutritional Status Test” (CONUT) to establish the nutritional status of the study patients^38^.

### Laboratory analysis

Blood samples were collected from all consenting patients at the time of the study visit. Blood samples were processed for plasma and then frozen at −80 °C until assayed. Plasma samples were analyzed for proinflammatory cytokines (interleukin-6 [IL-6], interleukin-8 [IL-8], interleukin-10 [IL-10], TNF-α, interleukin-1β [IL-1β], and monocyte chemoattractant protein-1 [MCP-1]) by multianalyte chemiluminescent detection using Multiplex kits (Millipore, Billerica, MA, USA) on a Luminex (Luminex, Austin, TX, USA) platform based on the manufacturer’s instructions. Plasma LPS and plasma LPS binding protein (LBP), and soluble CD14 (sCD14) were assessed using the Kinetic Chromogenic Limulus Amoebocyte Lysate Assay (Lonza, Walkersville, MD, USA) following the manufacturer’s instructions. Because portal vein sampling is invasive and not ethically feasible in early-stage clinical cohorts, gut-derived biomarkers were measured in systemic plasma samples. Circulating markers such as LPS, LBP, and sCD14 have been widely used as peripheral indicators of gut permeability and microbial translocation in ALD and reflect systemic exposure to gut-derived endotoxins.

### Proteomics analysis

Plasma peptides were extracted as recently described. Briefly, the plasma samples from the enrolled participants were aliquoted and diluted using an equal volume of K3-EDTA from BD Vacutainer® tubes. To precipitate proteins, 200 µL of ice-cold 20% w/v trichloroacetic acid (dissolved in LC-MS grade water) was added, and the samples were incubated for 5 min. This solution was vortexed and incubated on ice for 60 min to complete the plasma protein precipitation. Precipitated proteins and flocculated material were removed by two sequential centrifugation steps at 16,000 × *g*, 4°C, for 10 min. The low-molecular-weight proteome (also known as the peptidome), containing the supernatant, was transferred and consolidated into a clean tube at each successive clarification step. The peptidome was concentrated, desalted, and buffer-exchanged by solid-phase extraction using the manufacturer’s protocol with an Oasis HLB µElution plate (30 µm/2 mg, Waters, Milford, MA, USA) on a Presston 100 Positive Pressure Manifold (Phenomenex, Torrance, CA, USA). Using the flow rate of 1 mL/min with the loading buffer (5% v/v acetonitrile/0.1% v/v TFA) and elution buffers (40% v/v can/0.1% v/v TFA), the µelution plate (200 µl) was sequentially rinsed thrice. This sample was loaded and washed five times (200 µL) using the loading buffer and then eluted three times (50 µL) using the elution buffer. These elute samples were integrated and dried in a SpeedVac and then stored at −80 °C. The dried HLB eluates were subsequently resuspended in 25 µL 2% v/v acetonitrile/0.1% v/v formic acid. The peptide concentration was determined using a NanoDrop 2000 spectrophotometer (ThermoFisher Scientific, Waltham, MA, USA) by measuring the absorbance at 205 nm. Peptide aliquots were diluted to 0.1 µg/µL (by A205 absorbance), and 0.5× iRT retention time normalization standards (Biognosys, Schlieren, Switzerland) were added. Two microliters of each sample were analyzed using LC-MS/MS in a data-dependent manner^36^. Samples were loaded onto Acclaim PepMap 100, 75 μm × 2 cm, nanoViper (C18, 3 μm, 100 Å) trap and then eluted onto an Acclaim PepMap RSLC, 75 μm × 50 cm, nanoViper (C18, 2 μm, 100 Å) separating column (ThermoFisher) heated at 50 °C using an EASY-nLC 1000 UHPLC system (ThermoFisher) with a binary gradient (solvent A, 2% v/v acetonitrile/0.1% vol/vol formic acid; solvent B, 80% v/v acetonitrile/0.1% v/v formic acid). The peptidome was eluted using a 60-minute linear gradient from 0% to 55% B at a flow rate of 250 nL/min. Elutes were introduced into the MS with a 40 mm stainless steel emitter (ThermoFisher) positioned by a Nanospray Flex source (ThermoFisher) near the ion transfer capillary of the mass spectrometer. The ion transfer capillary temperature was set to 225 °C, and the spray voltage at 1.75 kV. At the end of the linear gradient for each sample elution, the column was recovered by a 5-minute linear gradient from 55% to 95% B, with a flow ramp from 250–300 nL/min. This was followed by a 10-minute wash with 95% B at 300 nL/min. LC-MS/MS data were collected using an Orbitrap Elite—ETD mass spectrometer (ThermoFisher). An Nth Order Double Play method was created in Xcalibur v2.2 (ThermoFisher) for data collection. Scan event 1 obtained an FTMS MS1 scan (normal mass range, 240,000 resolution, full scan type, positive polarity, and profile data type) for the range 300–2000 m/z. Scan event 2 obtained ITMS MS2 scans (normal mass range, rapid scan rate, and centroid data type) for up to 20 peaks with a minimum signal threshold of 5000 counts from scan event 1. The lock mass option was enabled (0% lock mass abundance) using a 371.101,236 m/z polysiloxane peak as an internal calibrant.

Raw files were searched in Peaks Studio X (Bioinformatics Solutions Inc., Waterloo, ON, Canada) using Denovo, PeaksDB, PeaksPTM, and Label-Free Q algorithms. The UniprotKB reference proteome canonical and isoform Homo sapiens sequences (Proteome ID UP000005640) version 11/30/2020 were used for human degradome samples, and the Mus musculus sequences (Proteome ID UP000000589) version 7/22/2021 were used for mouse degradome samples. Search parameters included: variable methionine and proline oxidation ( + 15.9949 Da), no enzyme specified, 15 ppm precursor error for MS1 Orbitrap FTMS data, 0.5 Da error for MS2 datasets, and selected common PTMs in the PeaksPTM algorithm. The peptide, areas from extracted ion chromatograms per feature, and protein-peptide level data were exported from the label-free Q result as CSV files or statistical tests in Microsoft Excel 2016 and R v3.5.0. High confidence peptide assignments (Peaks Studio X criteria –logP scores with FDR threshold 1%) were exported into comma-separated values files (.csv) as the total peptide areas from the extracted ion chromatograms for biostatistics and bioinformatic analyses. The accession numbers were refined through a series of steps, and MS data were processed using bioinformatics tools to match peptides to protein databases, enabling the identification of proteins and their respective peptides. Quantification was performed by comparing peptide ion intensities across samples, and the fold change was calculated by dividing the value of Group 2 by the value of Group 1. The log2 fold change was calculated as the base-2 logarithm of the fold change. The fold change greater than one indicates upregulation, whereas a fold change of less than one indicates downregulation, including combining significant entries, removing duplicates, and standardizing formats. Additional identifiers were removed, and the data were cleaned by eliminating rows with zero values across all groups^39^. This process resulted in a refined list of unique, standardized accession numbers representing peptides with significant inter-group differences.

Statistical analysis was performed to determine differential protein expression. *P*-values and adjusted *p* values (FDR corrected) were calculated to assess significance, and proteins were classified as significantly upregulated or downregulated based on thresholds (*P* < 0.05). Further annotations included protein accession numbers and peptide sequences.

The refined list of accession numbers was subjected to pathway analysis using protein analysis through the evolutionary relationships classification system^40^.

The mapped IDs were input into search tools to retrieve interacting genes and proteins and identify the involved molecules and their interactions^41^. String analysis was performed using the default parameters, with a medium confidence score threshold of 0.4.

Clinical and peptide columns were extracted from all subjects with complete data. Linear regression analysis was performed to evaluate associations between peptide abundance and clinical biomarkers. Multiple testing correction: FDR adjustment (Benjamini–Hochberg) was applied to all *p* values. Significance Threshold: FDR < 0.05 was used to define significant associations. Reactome-based protein interaction networks were constructed using experimentally validated interactions from the STRING database, with node positions determined by using a force-directed layout algorithm to optimize the spatial representation of interaction proximity. Functional annotations and pathway enrichment were mapped using node colors assigned by biological processes. Network topology metrics were calculated, and module detection was performed for structure identification. The statistical significance of the hub nodes and modules was assessed using permutation testing.

### Statistical analysis

All statistical analyses were performed using SPSS version 29.0 (IBM Corp., Armonk, NY, USA) and GraphPad Prism version 10.2.2 (GraphPad Software, Boston, MA, USA). Continuous variables are presented as mean ± standard deviation (SD) or median with interquartile range IQR, as appropriate. Due to the modest sample size and non-normal distribution of several variables, group comparisons were performed using nonparametric tests. Differences among groups were assessed using the Kruskal–Wallis test, followed by Dunn’s post hoc test for multiple comparisons where applicable. Associations between variables were evaluated using Spearman rank correlation analysis. Linear regression was applied for visualization and to estimate the strength of associations, with coefficients of determination *R*² reported. All statistical tests were two-tailed, and a *p* value < 0.05 was considered statistically significant.

## Data Availability

The datasets analyzed during the current study are available from the corresponding author upon reasonable request. No custom code was generated or used in this study; all analyses were performed using standard, publicly available software tools as described in the Methods section.

## Code availability

No custom code was used in this study. All analyses were performed using standard, publicly available software tools as described in the Methods section.
